# Mapping the human oral and gut fungal microbiota in patients with metabolic dysfunction-associated fatty liver disease

**DOI:** 10.3389/fcimb.2023.1157368

**Published:** 2023-04-26

**Authors:** Chenguang Niu, Ye Tu, Qiaoqiao Jin, Zhanyi Chen, Keyong Yuan, Min Wang, Pengfei Zhang, Junyuan Luo, Hao Li, Yueyi Yang, Xiaoyu Liu, Mengying Mao, Ting Dong, Wenduo Tan, Xuchen Hu, Yihuai Pan, Lili Hou, Rui Ma, Zhengwei Huang

**Affiliations:** ^1^ Department of Endodontics, Shanghai Ninth People’s Hospital, Shanghai Jiao Tong University School of Medicine, Shanghai, China; ^2^ College of Stomatology, Shanghai Jiao Tong University, Shanghai, China; ^3^ National Clinical Research Center for Oral Diseases, Shanghai, China; ^4^ National Center for Stomatology, Shanghai, China; ^5^ Shanghai Key Laboratory of Stomatology, Shanghai, China; ^6^ Institute of Stomatology, School and Hospital of Stomatology, Wenzhou Medical University, Wenzhou, China; ^7^ Department of Endodontics, School and Hospital of Stomatology, Wenzhou Medical University, Wenzhou, China; ^8^ Department of Nursing, Shanghai Ninth People’s Hospital, School of Medicine, Shanghai Jiaotong University, Shanghai, China

**Keywords:** metabolic dysfunction-associated fatty liver disease, oral mycobiome, gut mycobiome, oral-gut axis, metagenomics

## Abstract

Metabolic dysfunction-associated fatty liver disease (MAFLD) is a phenotype of liver diseases associated with metabolic syndrome. The pathogenesis MAFLD remains unclear. The liver maintains is located near the intestine and is physiologically interdependent with the intestine *via* metabolic exchange and microbial transmission, underpinning the recently proposed “oral-gut-liver axis” concept. However, little is known about the roles of commensal fungi in the disease development. This study aimed to characterize the alterations of oral and gut mycobiota and their roles in MAFLD. Twenty-one MAFLD participants and 20 healthy controls were enrolled. Metagenomics analyses of saliva, supragingival plaques, and feces revealed significant alterations in the gut fungal composition of MAFLD patients. Although no statistical difference was evident in the oral mycobiome diversity within MAFLD and healthy group, significantly decreased diversities were observed in fecal samples of MAFLD patients. The relative abundance of one salivary species, five supragingival species, and seven fecal species was significantly altered in MAFLD patients. Twenty-two salivary, 23 supragingival, and 22 fecal species were associated with clinical parameters. Concerning the different functions of fungal species, pathways involved in metabolic pathways, biosynthesis of secondary metabolites, microbial metabolism in diverse environments, and carbon metabolism were abundant both in the oral and gut mycobiomes. Moreover, different fungal contributions in core functions were observed between MAFLD patients and the healthy controls, especially in the supragingival plaque and fecal samples. Finally, correlation analysis between oral/gut mycobiome and clinical parameters identified correlations of certain fungal species in both oral and gut niches. Particularly, *Mucor ambiguus*, which was abundant both in saliva and feces, was positively correlated with body mass index, total cholesterol, low-density lipoprotein, alanine aminotransferase, and aspartate aminotransferase, providing evidence of a possible “oral-gut-liver” axis. The findings illustrate the potential correlation between core mycobiome and the development of MAFLD and could propose potential therapeutic strategies.

## 1 Introduction

Metabolic dysfunction-associated fatty liver disease (MAFLD) is a condition of fat accumulation in the liver in combination with obesity, type 2 diabetes, or metabolic dysfunction (Demir et al., 2022). Formerly known as nonalcoholic fatty liver disease, MAFLD has a global prevalence of 25%, and has surged to 29.2% in China’s mainland (Fan et al., 2021; Lu et al., 2022; Zhuge et al., 2022). The prevalence will continue to increase with the growing numbers of obese individuals with metabolic syndrome. The pathophysiology of MAFLD is complex and includes several mechanisms, with metabolic syndrome and insulin resistance playing major roles(Kuchay et al., 2020; Sakurai et al., 2021).

Accumulating evidence has indicated the role of gut microbiota in the development and progression of MAFLD, since the “gut-liver axis” was first proposed by Marshall in 1987(Volta et al., 1987). Given the direct connection between the intestine and the liver *via* the portal vein, the liver is more susceptible to gut microbiota, bacterial products, endotoxins, and microbiome inflammatory molecules(Jiang et al., 2020). Intestinal bacteria could migrate through the portal vein into the liver and lead to the abnormal activation of the immune system(Milosevic et al., 2019; Albillos et al., 2020). In turn, this would lead to an inflammatory response in patients with MAFLD. Most studies have focused on gut bacteria, with little attention given to fungal alterations(Heng et al., 2022).

The human gastrointestinal tract harboring a complex diversity of microorganisms, including bacteria, fungi, viruses, protozoa, and archaea. Fungi constitute only approximately 0.1% of the human gut microbiome(Huffnagle and Noverr, 2013). Yet, they are an indispensable part of this microbiome and have vital roles in multiple physiological processes. The oral cavity is the second-largest microbial community in the human body(Pathak et al., 2021). It is among the most diverse microbial ecosystems in the human body, together with the gut, harboring over 600 bacterial species and 100 fungal species(Fidel et al., 2021). Recent studies have supported a possible pathogenic impact of the oral microbiome and oral disease on gastrointestinal and liver diseases. For example, preclinical and clinical research have shown periodontitis, a common periodontal disease initiated by oral microbial dysbiosis, can exacerbate MAFLD(Albuquerque Souza and Sahingur, 2022; Wang et al., 2022).

The oral microorganisms vary across niches, such as dental plaque, tongue, saliva, and gingival sulcus. Oral microorganisms could translocate to the gut and further disseminate from the gut lumen to the liver *via* the portal vein and cause liver disease(Park et al., 2021; Zhang et al., 2022). Since the gastrointestinal tract receives a constant flow from mouth to anus, it is possible that the microbiota or their by-products in the oral cavity disseminate to the liver after the oral-gut microbial translocation, and thus contribute to the pathogenesis of liver diseases. For example, *Porphyromonas gingivalis*, which causes periodontitis, is associated with liver inflammation(Albuquerque Souza and Sahingur, 2022; Wang et al., 2022). However, the oral fungal microbiome has received much less attention, albeit its ecological and clinical significance.

This study is the first characterization of the oral and gut fungal microbiomes using metagenomics sequencing in patients diagnosed with MAFLD. The fungal composition of oral and gut niches are revealed. Alterations of oral and gut mycobiota between MAFLD and healthy controls were investigated. Correlations between oral/gut mycobiota and clinical parameters were assessed to characterize MAFLD-related fungi. Functional analyses were performed. The possible link between oral-gut *Mucor ambiguus* that was evident suggests the microbial transmission between oral and gut niches in the pathogenesis of MAFLD.

## 2 Methods

### 2.1 Study participants

This study followed the Declaration of Helsinki on medical protocols and ethics, and was approved by the Ethics Committee of School and Stomatology Wenzhou Medical University (approval no. WYKQ2021006). Twenty-one MAFLD patients who met the inclusion and exclusion criteria were enrolled in the study from July 2021 to June 2022. Twenty healthy controls were enrolled by matching age. Inclusion criteria were age over 18 years and clinically proven MAFLD according to the Asian Pacific Association for the Study of the Liver guidelines. The exclusion criteria were as follows: (1) presence of other liver diseases, including viral hepatitis, autoimmune hepatitis, and hepatolenticular degeneration; (2) hepatic steatosis induced by drugs, such as tamoxifen, amiodarone, valproate, methotrexate, and glucocorticoids; (3) other factors that may cause hepatic steatosis, including long-term total parenteral nutrition, inflammatory bowel disease, celiac disease, hypothyroidism, Cushing’s syndrome, lipoprotein deficiency, lipid-atrophic diabetes, and others; (4) use of lipid-lowering drugs in the 6 months preceding enrollment; (5) type 1 or type 2 diabetes; (6) current oral diseases, including untreated oral abscess, precancerous lesions, oral cancer, oral fungal infection, missing more than eight teeth, periodontitis and others; (7) use of probiotics, antifungal drugs; and (8) other conditions, including pregnant or lactating women, long-term heavy smoking, use of antibiotics for more than 5 days within the preceding 6 months, severe acute episode of a systematic disease, abnormal thyroid function, familial hyperlipidemia, and others. Written informed consent was obtained from all participants prior to enrollment. Patient characteristics are summarized in **Table 1**.

**Table 1:** Clinical characteristics of the study cohort.

Characteristics	Control (n=20)	MAFLD (n=21)	*P* value
Sex ( M/F )	16-Apr	16-May	1
Age ( years )	29.00 ± 8.89	32.43 ± 6.59	0.1778
Height ( cm )	172.10 ± 7.59	169.33 ± 6.71	0.2344
Weight ( kg )	65.68 ± 8.55	74.81 ± 13.08	0.0142*
BMI ( kg/m2 )	22.09 ± 1.92	25.96 ± 3.27	0.0001*
TC ( mmol/L )	4.30 ± 0.64	4.87 ± 1.02	0.0429*
TG ( mmol/L )	1.07 ± 0.44	1.82 ± 0.82	0.0010*
LDL-C ( mmol/L )	2.59 ± 0.50	3.13 ± 0.91	0.0291*
HDL-C ( mmol/L )	1.25 ± 0.24	1.09 ± 0.23	0.0395*
GGT ( U/L )	16.40 ± 4.98	35.10 ± 18.93	0.0002*
AST ( U/L )	18.10 ± 5.13	28.33 ± 20.88	0.0443*
ALT ( U/L )	17.05 ± 9.13	39.33 ± 23.32	0.0004*
FBG ( mmol/L )	4.71 ± 0.33	4.85 ± 0.44	0.2652
FSI ( mU/L )	5.90 ± 2.18	11.30 ± 5.20	0.0002*
HOMA-IR	1.28 ± 0.48	2.47 ± 1.27	0.0003*
HbA1c	5.18 ± 0.21	5.33 ± 0.28	0.0664
CRP ( mg/L )	1.15 ± 1.28	2.11 ± 2.38	0.1296
WBC ( ´ 109/L)	5.90 ± 1.12	6.28 ± 1.25	0.3282

BMI, body mass index; TC, total cholesterol; TG, total triglyceride; LDL-C, low-density lipoprotein cholesterol; HDL-C, high-density lipoprotein cholesterol; ALT, alanine aminotransferase; AST, aspartate aminotransferase; GGT, gamma glutamyl transpeptidase; FBG, fasting blood glucose; FSI, fasting serum insulin; HbA1c, glycated hemoglobin A1c; WBC, white blood cell; CRP, plasma high-sensitivity C-reactive protein; IR, insulin resistance; HOMA-IR, the Homeostatic Model Assessment for Insulin Resistance. Values are presented as mean and standard deviation. Student’s t-test or the Kruskal-Wallis test was applied for analysis of all clinical variables. *P < 0.05.

### 2.2 Acquisition of clinical variables

Demographic information of all patients, including weight, height and dietary habits, were obtained by trained staff in Wenzhou Medical University. Clinical parameters were collected with hematologic examination after 12 h of fasting. These parameters included total cholesterol (TC), total triglyceride (TG), low-density lipoprotein cholesterol (LDL-C), high-density lipoprotein cholesterol (HDL-C), alanine aminotransferase (ALT), aspartate aminotransferase (AST), gamma glutamyl transpeptidase (GGT), fasting blood glucose (FBG), fasting serum insulin (FSI), glycated hemoglobin A1c (HbA1c), white blood cell (WBC) and plasma high-sensitivity C-reactive protein (CRP). As an approximation of insulin resistance (IR), the Homeostatic Model Assessment for Insulin Resistance (HOMA-IR) score was calculated as [FPG (mmol/L) × FSI (mU/L)]/22.5.

### 2.3 Sample collection

A total of 123 samples (41 saliva, 41 supragingival plaque, and 41 feces samples) were collected from the 41 participants as previously described(Zhao et al., 2020; Chen et al., 2022). Briefly, each participant were required to rinse their mouth and avoid eating and drinking for at least 1 h before collection of the oral sample. Saliva was collected and preserved in 50 mL sterile tubes (Corning, New York, NY, USA) containing saliva DNA preservation solution (Huayueyang Biotech, Beijing, China). Supragingival plaque was collected before eating in the morning in accordance with the methods described in the Manual of Procedures for the Human Microbiome Project. The fecal sample from each participant was freshly collected in specially provided collection containers. Each fecal sample was transported to the laboratory in a box containing an ice pack within 2 h of collection and stored at -80°C. Whole-blood samples were collected in tubes with anticoagulants (Improve Medical, Guangzhou, China) after at least 8 h of fasting.

### 2.4 DNA extraction and metagenomic sequencing

Total bacterial genomic DNA was extracted from the collected supragingival samples using the QIAamp DNA Mini Kit (Qiagen, Valencia, CA, USA) in accordance with manufacturer’s protocols. The concentration and purification of the extracted DNA were determined using a NanoDrop 2000 UV-vis spectrophotometer (Thermo Fisher Scientific, Waltham, MA, USA). DNA quality was checked by 1% agarose gel electrophoresis.

The extracted DNA was fragmented to an average size of approximately 400 bp using a Covaris M220 ultrasonicator (Gene Company Limited, Huzhou, China) for paired-end library construction. The paired-end library was constructed using NEXTFLEX^®^ Rapid DNA-Seq (Bioo Scientific, Austin, TX, USA). Adapters containing the full complement of sequencing primer hybridization sites were ligated to the blunt-end of fragments. Paired-end sequencing was performed using a Novaseq 6000 device (Illumina Inc., San Diego, CA, USA) at Majorbio Bio-Pharm Technology Co., Ltd. (Shanghai, China) using NovaSeq Reagent Kits according to the manufacturer’s instructions. The data were analyzed on the free online Majorbio Cloud Platform. Briefly, the paired-end Illumina reads were trimmed of adaptors, and low-quality reads (length<50 bp or with a quality value <20 or having N bases) were removed by fastp (https://github.com/OpenGene/fastp, version 0.20.0).

### 2.5 Gene prediction, taxonomy, and functional annotation

Open reading frames (ORFs) from each assembled contig were predicted using Prodigal(Hyatt et al., 2010) /MetaGene(Noguchi et al., 2006). The predicted ORFs with a length ≥ 100 bp were retrieved and translated into amino acid sequences using the NCBI translation table.

A non-redundant gene catalog was constructed using CD-HIT(Fu et al., 2012) with 90% sequence identity and 90% coverage. High-quality reads were aligned to the non-redundant gene catalogs to calculate gene abundance with 95% identity using SOAPaligner.

Representative sequences of non-redundant gene catalog were aligned to NR database with an e-value cutoff of 1e-5 using Diamond for taxonomic annotations. Cluster of orthologous groups of proteins (COG) annotation for the representative sequences was performed using Diamond against eggNOG database with an e-value cutoff of 1e^-5^. Kyoto Encyclopedia of Genes and Genomes (KEGG) annotation was performed using Diamond against the KEGG database with an e-value cutoff of 1e^-5^.

### 2.6 Statistical analyses

The clinical variables are demonstrated by counts. Chi-squared test was used for differential analyses. Continuous variables are presented as mean ± standard deviation. Student’s *t-*test or the Kruskal-Wallis test was applied for analysis of all clinical variables. A *P*-value < 0.05 was considered significant. Alpha diversity was calculated using the Shannon, Simpson, and Chao-1 indices. The equality of variance was confirmed with Levene’s test. Normality of the data was evaluated using the Shapiro–Wilk test. The significance of genotype was assessed with the Student’s *t* test. Beta diversity was assessed by the principal coordinate analysis (PCoA) with Bray–Curtis distance used to calculate the distance metric. Analysis of similarities (ANOSIM) test was used for the statistical analysis. The Kruskal–Wallis test was used to detect significant differences in abundance, and the Wilcoxon rank-sum test was used for *post hoc* comparison. A *P*-value < 0.05 was considered significant. Linear discriminant analysis effect size (LEfSe) was used to identify the significant differential fungal biomarkers with a linear discriminant analysis (LDA) score > 2.0. Correlation analysis was constructed to investigate the interaction between fungal species and clinical parameters, and Spearman coefficient |r| > 0.5 and *P* < 0.05 are shown. Spearman’s correlations of fungal abundance among different sample sites were tested with a coefficient |r| > 0.5 and *P* < 0.05. P < 0.05 is marked with *, *P* < 0.01 with ** and *P* < 0.001 with ***.

## 3 Results

### 3.1 Study population and clinical characteristics

A total number of 41 participants were recruited for the study. Twenty-one had been diagnosed with MAFLD and 20 were healthy controls (**Table 1**). The two confounding factors of age and gender were matched at the time of inclusion of the study subjects. Overall, there were no significant differences between the two groups concerning age and gender. In the absence of significant differences in height between the two groups, the MAFLD group had significantly higher body weight and correspondingly higher body mass index (BMI) than the control group. Compared to the control group, the MAFLD group had lower HDL-C levels and higher levels of TC, TG, LDL-C, GGT, AST, and ALT. There was no difference in FBG between the two groups, but FSI was relatively higher in the metabolic group, resulting in a higher HOMA-IR in the MAFLD group than in the control group.

### 3.2 Fungal diversity of oral and gut mycobiome in MAFLD patients

**Figure 1:**
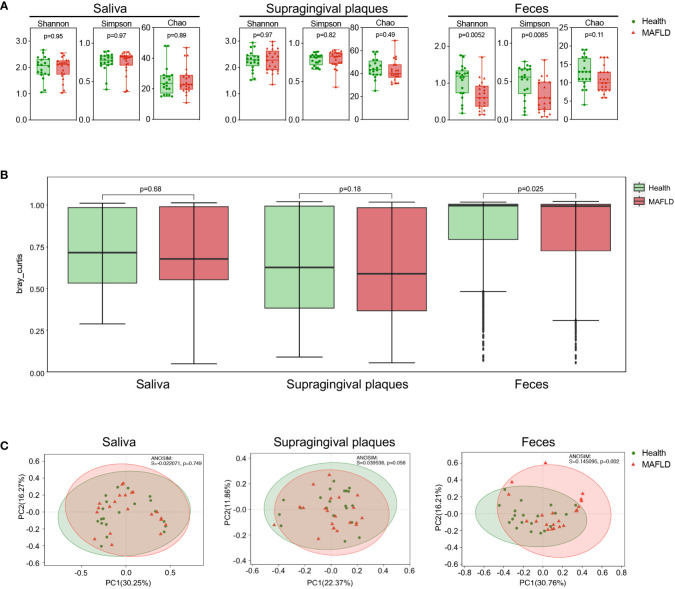
Fungal diversity of oral and gut mycobiome in MAFLD patients. **(A)** Shannon, Simpson, and Chao1 indices of the oral (saliva and supragingival plaques) mycobiome and gut (feces) mycobiome between MAFLD patients and healthy controls (*t* test). **(B)** Bray–Curtis distance of the beta-diversity in the oral and gut mycobiome in MAFLD patients and healthy controls. **(C)** Principal coordinate analysis (PCoA) plots and analysis of similarities (ANOSIM) statistical data of the oral and gut mycobiome between MAFLD patients and healthy controls. *P* < 0.05 with Wilcoxon rank-sum test followed by Kruskal-Wallis test.

To investigate the mycobiome profile of two groups, metagenomic sequencing was performed on the oral (saliva and supragingival plaques) and gut (feces) samples from the 41 participants. Alpha diversity analysis demonstrated no significant difference in the fungal diversity (Shannon and Simpson indices) and fungal richness (Chao index) of the oral mycobiome between the MAFLD patients and healthy controls. Lower alpha diversity was observed with the fecal mycobiome in the MAFLD patients, as indicated by the significantly decreased Shannon and Simpson indices (*P* = 0.0052 and 0.0085, respectively; (**Figure 1A**). PCoA plots were measured by Bray-Curtis distance of the three sample types **Figure 1B**). ANOSIM revealed the significant difference of the gut fungal structure between MAFLD patients and the healthy controls (*P* = 0.002). Little difference was observed with regard to the oral mycobiome, although a statistical tendency (*P* = 0.056) was detected in the suragingival plaques (**Figure 1C**).

### 3.3 Composition of oral and gut mycobiome in MAFLD patients

**Figure 2:**
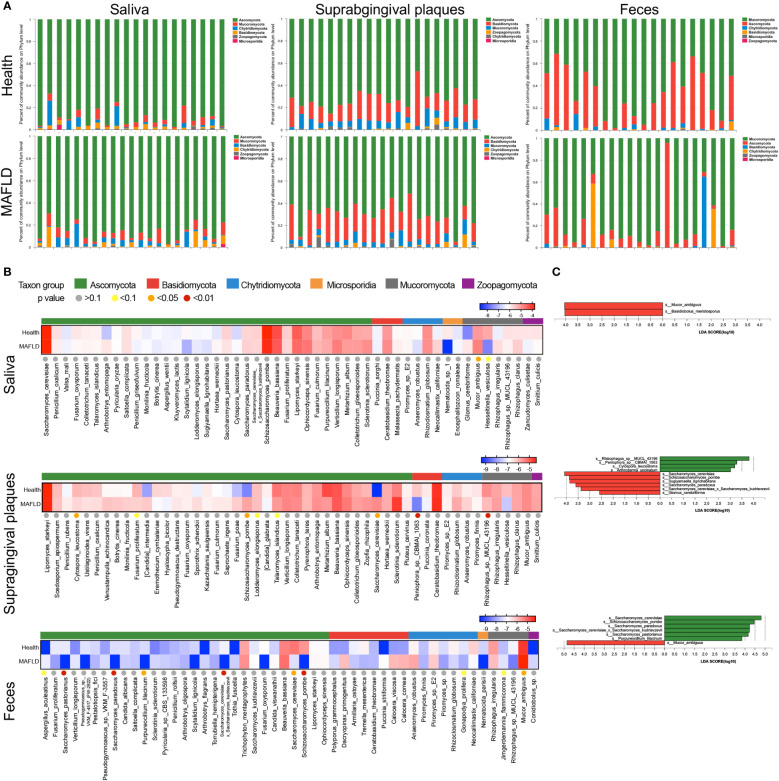
Compositional alterations of oral and gut mycobiome between MAFLD patients and healthy controls. **(A)** Relative abundance of oral (oral and supragingival plaques) and gut (feces) mycobiota at the phylum level. **(B)** Clustering heatmaps of the common logarithm of abundance of the top 50 species. The color of dots reflects the *P*-value of the differential analysis. **(C)** Differential fungal species in the saliva, supragingival mycobiome and gut mycobiome between MAFLD patients and healthy controls.

The oral and gut fungal community of the current cohort mainly consisted of six phyla, including *Ascomycota*, *Basidiomycota*, *Chytridiomycota*, *Microsporidia*, *Mucoromycota*, and *Zoopagomycota* (**Figure 2A**). The top 50 most abundant fungal species of three loci were then identified. Seven species in feces, five in supragingival plaques, and one in saliva presented significantly different abundance between the MAFLD and control groups (*P* < 0.05, **Figure 2B**). To further identify the significantly enriched fungal species in MAFLD patients, LEfSe was used to identify fungi that differed significantly between the MAFLD and control groups. Species with LDA scores ≥ 2.0 were confirmed and are shown in **Figure 2C**. Focusing on the top 50 species, *Mucor ambiguous* (*M. ambiguous*), a member of the *Mucoromycota* phylum, was enriched in both saliva and feces in MAFLD patients. *Saccharomyces cerevisiae* (*S. cerevisiae*) and *Schizosaccharomyces pombe* (*S. pombe*), were more abundant in supragingival plaques of MAFLD groups. Conversely, these two species were more abundant in feces of the healthy group.

### 3.4 Correlation analysis of oral/gut mycobiome and clinical indicators

**Figure 3:**
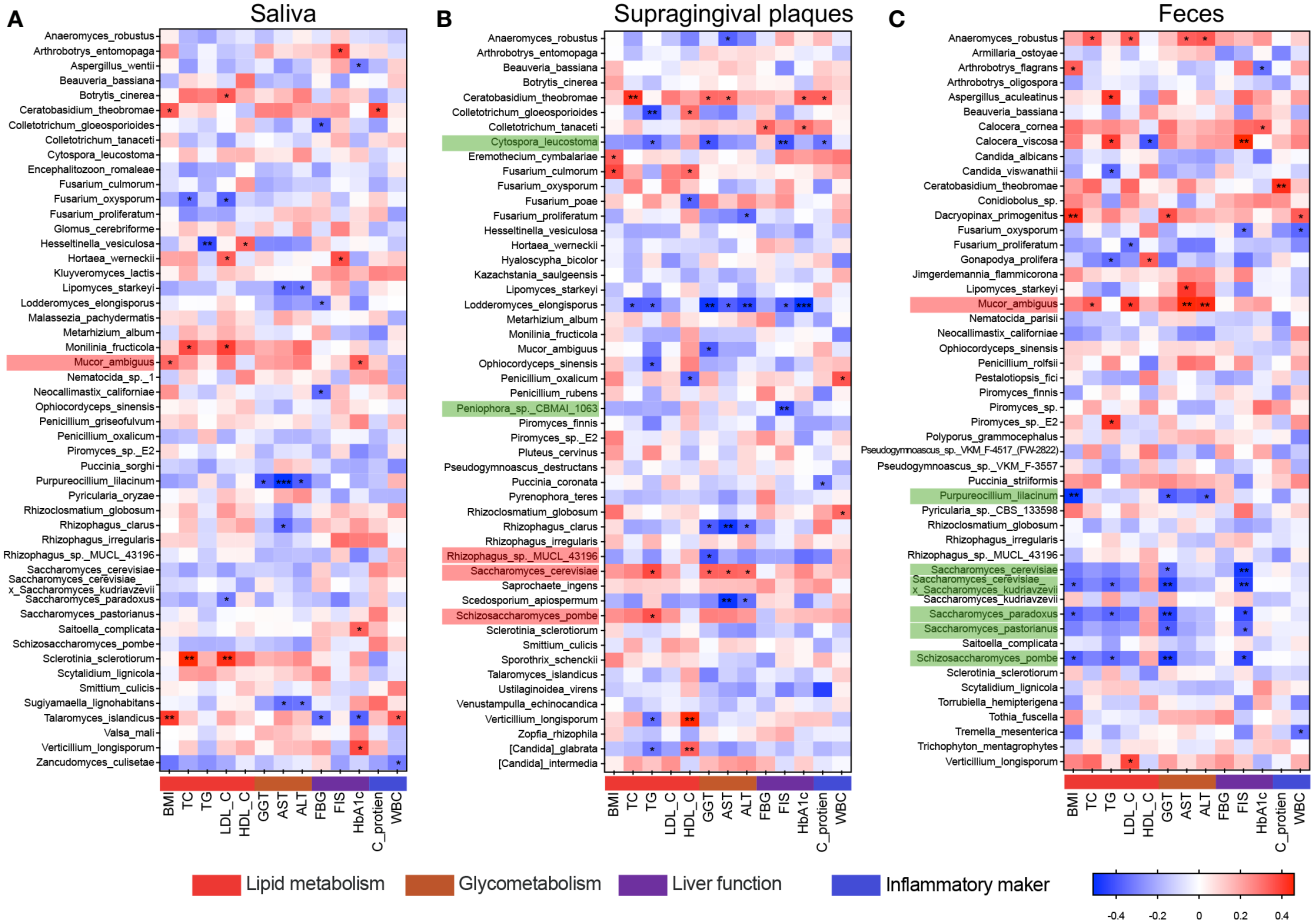
Correlation analysis of oral/gut mycobiome and clinical indicators. Heatmaps of Spearman correlation coefficients between clinical indicators and the top 50 species in saliva **(A)**, supragingival plaques **(B)**, and feces **(C)**. Species highlighted in red and green are those significantly enriched in MAFLD patients and healthy controls, respectively. BMI, body mass index; TC, serum total cholesterol; TG, triglyceride; LDL-C, low-density lipoprotein cholesterol; HDL-C, high-density lipoprotein cholesterol; GGT, gamma-glutamyl transferase; AST, aspartate transaminase; ALT, alanine transaminase; FBG, fasting blood glucose; FSI, fasting serum insulin; HbA1C, glycosylated hemoglobin type A1C; CRP, C-reactive protein; WBC, white blood cells. *P (FDR) < 0.05.

To evaluate the clinical significance of the prevailing fungal species, Spearman correlation analyses were performed to assess the associations between the clinical indicators and the top 50 species, focusing on distinct species among three loci (**Figure 3**). Among the top 50 species in oral and gut samples, significant associations with various clinical parameters were evident for 22 species in saliva, 23 species in supragingival plaques, and 22 species in feces (**Figures 3A, B, C**). Significantly enriched fungal species within the top 50 species in the MAFLD and healthy groups were separately highlighted in red and green in the figure. Significantly enriched in the saliva of MAFLD patients, *M. ambiguus* was positively associated with BMI and HbA1c (*P* < 0.05, **Figure 3A**). Simultaneously presenting higher abundance in the feces of MAFLD patients, *M. ambiguus* was positively correlated with TC (*P* < 0.05), LDL-C (*P* < 0.05), ALT (*P* < 0.01), and AST (*P* < 0.01) (**Figure 3C**). Being enriched in the feces of healthy subjects, *S. cerevisiae* was negatively correlated with GGT (*P* < 0.05) and FSI (*P* < 0.01, **Figure 3C**). *S. pombe* in supragingival plaques was positively associated with TG (*P* < 0.05), whereas *S. pombe* was negatively correlated with TG (*P* < 0.05), BMI (*P* < 0.05), FSI (*P* < 0.05), and GGT (*P* < 0.01) in feces.

### 3.5 Functional differences of oral/gut mycobiome in MAFLD patients

**Figure 4:**
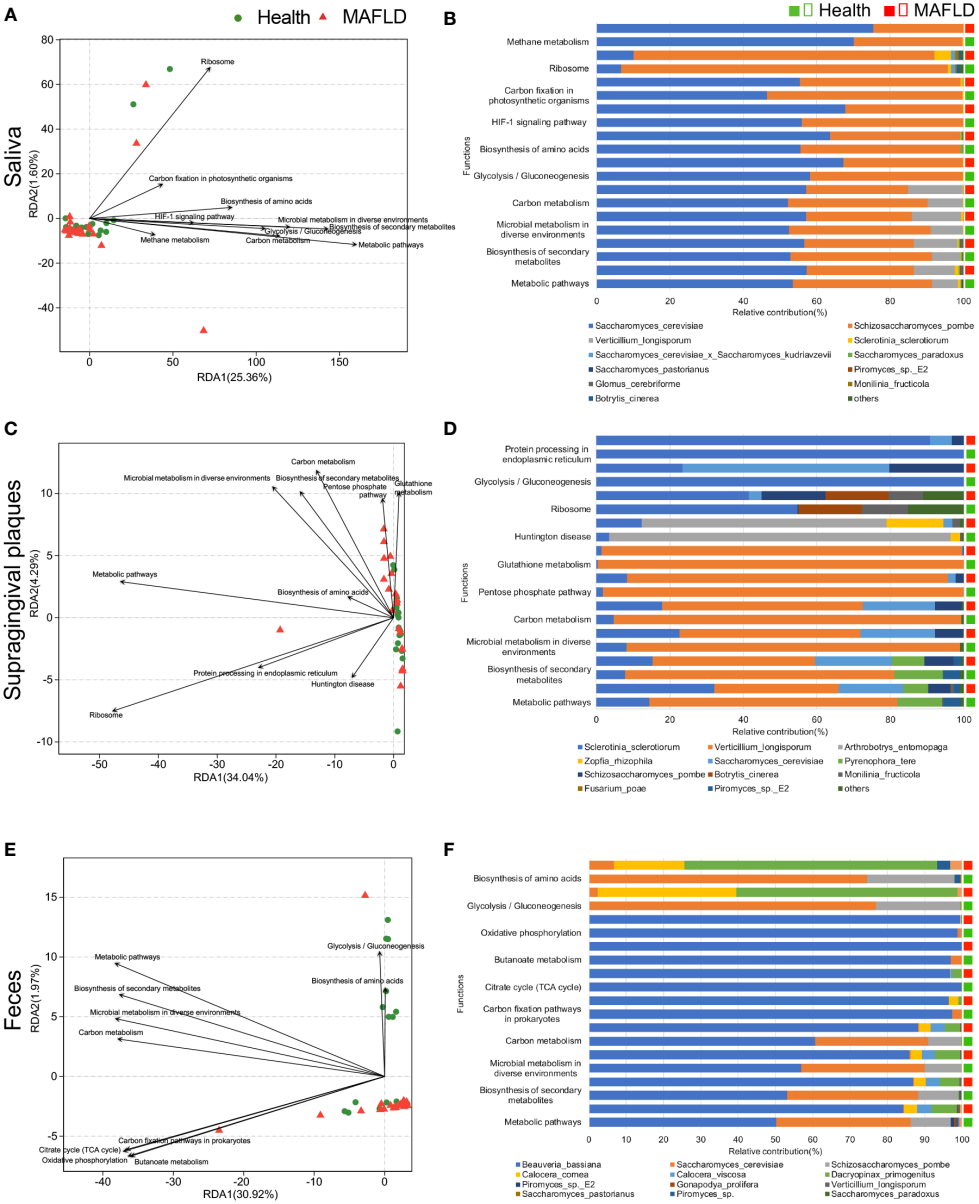
Functional differences of the oral/gut mycobiome in MAFLD patients. The relationship between clinical indicators and KEGG pathway in saliva **(A)**, supragingival plaques **(C)**, and feces **(E)**. The different colors and shapes of the points represent different groups, and the distance between the points represents the similarity and difference of the functional composition between the samples. The length of the arrow line represents the degree of correlation between clinical indicators and the KEGG pathway distribution (the longer the line, the greater the correlation). Species contribution analysis for the above 10 KEGG pathways in saliva **(B)**, supragingival plaques **(D)**, and feces **(F)**. The vertical axis represents the KEGG pathways, and the horizontal axis represents the proportion of different fungal species.

To elucidate functional profiles of mycobiome in saliva, supragingival plaques, and feces, gene were annotated using the KEGG database. Redundancy analysis (RDA) revealed the top 10 functional pathways at KEGG level 3 related to clinical indicators. Pathways involved in metabolic pathways, biosynthesis of secondary metabolites, microbial metabolism in diverse environments and carbon metabolism were of high abundant in all loci, suggesting that these are core functions (**Figures 4A, C, E**). The contributions of prevalent fungi on the top 10 functional pathways were further analyzed. In saliva, *S. cerevisiae* and *S. pombe* were the main contributors of the top 10 pathways, and slight contribution difference of each pathway was observed between MAFLD patients and healthy controls (**Figure 4B**). In supragingival plaques, different taxa contribution was observed in several disease-related pathways, including glycolysis/gluconeogenesis, carbon metabolism, microbial metabolism, biosynthesis of secondary metabolites, and metabolic pathways, and contributions of *S. cerevisiae* to these pathways were significantly higher in the MAFLD group than the healthy group (**Figure 4D**). In feces however, less contributions of *S. cerevisiae* to these pathways were identified in MAFLD patients (**Figure 4F**). Among the top 3 abundant functions, the taxa contribution was also significantly different between the MAFLD patients and healthy controls, particularly for supragingival plaques and feces. Network analysis indicated an increased number of taxa that involved in core functions from healthy group to MAFLD group (**Figure S1**).

### 3.6 Correlation analysis of oral and gut mycobiome in MAFLD patients

**Figure 5:**
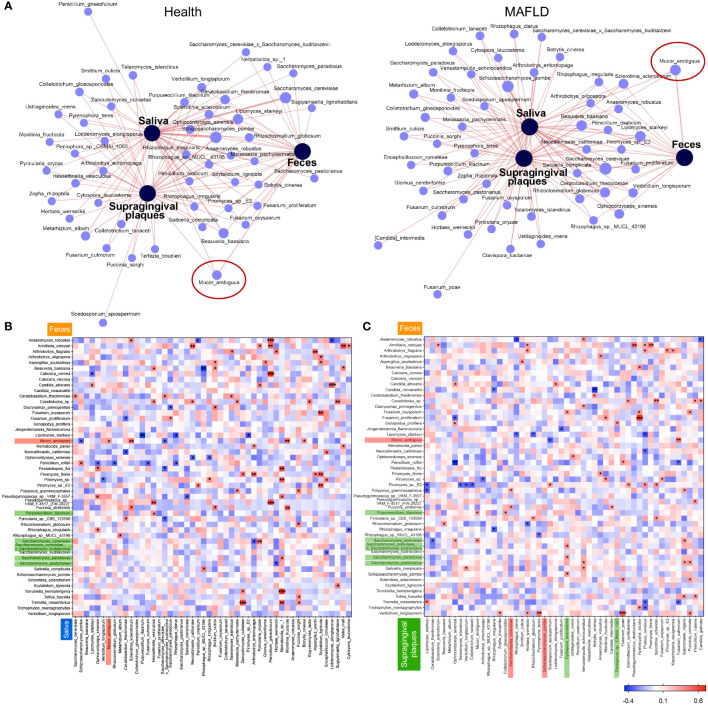
Communications between oral and gut microbiota in participants with or without MAFLD. Co-occurrence network analysis of top 50 fungal species among the three loci in healthy controls and MAFLD patients **(A)**. Heatmaps of Spearman’s correlation coefficients between relative abundances of shared genera in salivary **(B)** or supragingival **(C)** microbiota and those in feces. *P < 0.05, **P < 0.01, ***P < 0.001.

The gut-transiting oral microorganism has been linked to human health. Whether this is also the case for MAFLD was investigated here. Among the top 50 species in healthy individuals, 49 fungal species in saliva, 43 in supragingival plaques, and 28 in feces were identified, with 22 shared species in all three loci (**Figure S2**). In MAFLD patients, 48 species in saliva, 50 in supragingival plaques, and 18 in feces were identified, with 18 shared species (**Figure S2**). The distribution of the top 50 fungal species at the three loci differed significantly between MAFLD patients and healthy subjects (**Figure 5A**). To understand the potential oral-gut connection, Spearman’s correlation analysis based on the taxa relative abundance was performed. Among the top 50 species, there were 100 significant correlations between the feces and saliva and 95 significant correlations between the feces and supragingival plaques. *M. ambiguus* was found in all three loci in MAFLD patients (**Figure 5A**). In addition, the abundance of *M. ambiguous* in feces was positively correlated with its abundance in saliva (**Figure 5B**). The findings suggest that the potential ectopic colonization of *M. ambiguus* was more likely involved in the pathogenesis and progression of MAFLD. The decrease of *Cytospora leucostoma* in supragingival plaques was also positively correlated with the decrease of *S. paradoxus* and *Saccharomyces_cerevisiae_x_Saccharomyces_kudriavzevii* in feces (**Figure 5C**). These three species were both enriched in heathy controls and decreased in MAFLD patients, suggesting the beneficial roles in health.

**Figure 6:**
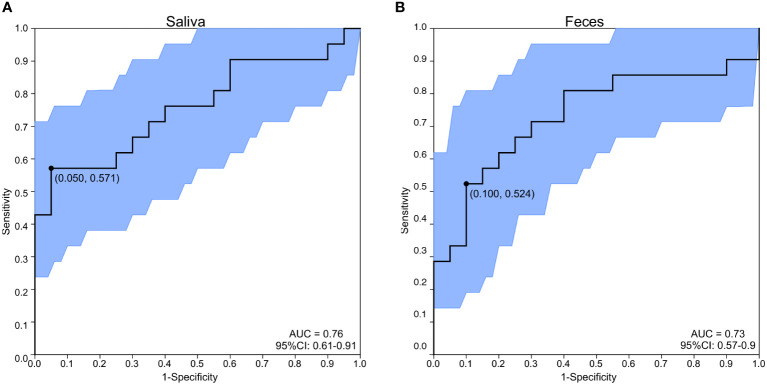
ROC analysis of *M. ambiguus* as a marker for MAFLD. ROC analysis of *M. ambiguus* in saliva **(A)** and feces **(B)**. The area under the ROC curve (AUC) of *M. ambiguus* classification. The black bars denote the 95% confidence interval (CI) and the area between the two outside curves represents the 95% CI shape.

To further identify the import role of *M. ambiguus*, receiver operating characteristic (ROC) analysis was performed to explore whether *M. ambiguus* can be used as a microbial marker of MAFLD. The area under the ROC curve were 0.76 and 0.73 in saliva and feces, respectively (**Figures 6A, B**), implicating salivary *M. ambiguus* as being potential for the diagnosis of MAFLD.

## 4 Discussion

Numerous studies have addressed the involvement of the bacterial microbiome in the development of MAFLD(Oh et al., 2020; Drozdz et al., 2021; Rao et al., 2021). However, knowledge of the fungal microbiome and MAFLD remains limited. The fungal community is a prominent component of the oral microbiome(Baker et al., 2017), yet the mycobiome has been much less studied compared to the bacterial community at any anatomical site, including the oral cavity. In this study, the fungal microbiome from two niches (oral and gut) with three different types of samples (saliva, supragingival plaques, and feces) in a cohort of MAFLD participants and healthy controls was investigated, and the functions in different niches was further illustrated. Considering that the patients’ gender is one of the main factors affecting the composition of gut microbiota(Fransen et al., 2017; Pepoyan et al., 2018), a non-parametric analysis of the gender of the recruited patients was performed. There was no difference in gender between the two groups in the present study, which also removed the confounding factors introduced by gender. The redesignation of MAFLD from NAFLD reflects the demonstrated involvements of metabolic alterations and metabolism-related pathways, such as biosynthesis of amino acids, carbon metabolism, and biosynthesis of secondary metabolites.

The richness of the gut mycobiome showed a similar tendency in MAFLD patients, and the community diversity was significantly decreased in MAFLD patients compared to healthy individuals. Both alpha and beta diversities were barely changed in the oral mycobiome, although reduced diversity was evident in the supragingival plaques of MAFLD patients compared with the healthy controls. *Ascomycota* and *Basidiomycota* constituted the two main predominant phyla in the oral mycobiome in both MAFLD patients and healthy controls. This finding is consistent with previous descriptions of proportions of *Ascomycota* and *Basidiomycota* of 75.5% and 24.5%, respectively, from 304 healthy individuals. The fungal composition of the plaques differed from the saliva samples, with a lower proportion of *Ascomycota*, and a higher proportion of *Basidiomycota*. As the initial site of the gastrointestinal tract, the oral niche features some special variables not present in feces, such as oral hygiene, oral health (bleeding gums, mouth ulcer, and false teeth), and others. The human mouth harbors a range of substrates, including teeth, tongue, cheeks and gums. Each habitat supports a complex, distinctive community(Mark et al., 2020). *S. cerevisiae* is the most abundant fungal species in the saliva. It was observed no significant difference in the prevalence of *S. cerevisiae* between MAFLD patients the healthy controls. Moreover, a higher proportion of *S. cerevisiae* was observed in the supragingival plaques of MAFLD patients and the gut mycobiome of the healthy controls. Though the tendency was independent in the two different niches, *S. cerevisiae* remained the biomarker species according to the LEfSe results. *S. cerevisiae* is one of the most concerned microbial species used for industrial production and is an important model organism to understand the biology of the eukaryotic cells and humans(Liu et al., 2021). A study followed 298 pairs of healthy mothers and offspring from 36 weeks of gestation until 2 years of age to explore the gut fungi in maternal and offspring samples(Schei et al., 2017). The authors detected very few *S. cerevisiae* in offspring at 10 days and 3 months after birth. However, once solid food was introduced to the diet, *S. cerevisiae* became the dominant species(Schei et al., 2017). Another study investigating the gut mycobiome variations across geography, ethnicity and urbanization, demonstrated a high enrichment of *S. cerevisiae* in urban populations compared with rural populations, with a significant inverse correlation with liver pathology-associated blood parameters, including AST, ALT, GGT, and direct bilirubin, indicating that *S. cerevisiae* may protect against liver-injury associated diseases(Sun et al., 2021). Concordant findings were observed in the present study, presenting inverse correlations between fecal *S. cerevisiae* and GGT and FIS

As for the significant biomarker, *M. ambiguus* was enriched in saliva and fecal samples of MAFLD group. *Mucor* sp. are common soil fungi but also known as agents of human infections (mucormycosis) and used in food production and biotechnology(Petrikkos et al., 2012; Walther et al., 2013; Wagner et al., 2020). These organisms can increase the intestinal permeability in epithelial cell monolayers(Mueller et al., 2019). It was observed a positive association between *M. ambiguus* and some clinical parameters, including BMI, HbA1c, TC, LDL-C, ALT and AST. Moreover, the log-ratios were of interest, given the findings of previous studies indicating that the log-ratios of *Mucor* sp./*S. cerevisiae* and *Candida albicans*/*S. cerevisiae* were independently associated with higher inflammatory activity. Munevver et al. reported a significant higher log-ratio of *Mucor* sp./*S. cerevisiae* in patients with non-alcoholic steatohepatitis, which also associated with serum glucose levels, AST levels, stage of fibrosis, and grade of liver inflammation(Demir et al., 2022). A random forest model indicated a predictive effect of *M. ambiguus* in saliva and feces for MAFLD, which might suggest an oral-gut axis in the development of MAFLD. Preclinical and clinical studies have shown the relationship between oral diseases and systematic diseases(Farrell et al., 2012; Ramos-Garcia et al., 2021). Although defined as two different niches in healthy state, some oral microbiota can translocated to the gut, where they cause microbial dysbiosis and disrupt intestinal permeability, further aggravating systematic inflammation. Oral fungi are expected to be biomarkers for the early diagnosis of many diseases and have great clinical applications. Compared to the intestinal ROC, greater ROC of oral *Mucor* was observed in the current project, suggesting that oral *Mucor* can served as a potential biomarker for the MAFLD diagnosis.

Limitations of this study include the lack of data on oral hygiene and dental status of the participants, which restricted the correlation between the oral health and clinical parameters of MAFLD. Moreover, since *M. ambiguus* was a significant biomarker in both the oral and gut mycobiomes, its role and effects should be further investigated with *in vitro* and *in vivo*.

In conclusion, the present study provides the first data of the composition and function of oral mycobiome and gut mycobiome in MAFLD patients. A strong correlation between *S. cerevisiae* and MAFLD-associated clinical parameters was found. The findings indicate a potential oral-gut communication of *M. ambiguus.* These data reinforce the role of fungal microbiota in the development of MAFLD, and strengthen the correlation of the oral-gut axis in metabolic diseases.

## Data availability statement

The data presented in the study are deposited in the NCBI sequencing read archive repository, accession number SRP429021.

## Ethics statement

The studies involving human participants were reviewed and approved by Ethics Committee of School and Stomatology Wenzhou Medical University. The patients/participants provided their written informed consent to participate in this study.

## Author contributions

ZH, RM and YP designed the study. CN, MW, PZ, XL, XH and YY collected clinical samples and conducted experiments. YT, ZC, WT, TD, MM, QJ and KY analysed the data. CN and YT prepared manuscript. ZH, HL, LH and JL revised the manuscript. All authors contributed to the article and approved the submitted version.

## Supplementary material

Supplementary Figure 1 Correlation analysis between function and fungal species of healthy controls and MAFLD patients. Correlation network analysis between functional alterations and differential fungal species. Spearman correlation > 0.5 or ≤ 0.5 is represented.

Supplementary Figure 2 Shared species in participants with or without MAFLD. Venn diagrams showing the unique and shared species among saliva, supragingival plaques, and feces. The 50 most abundant species were used for the Venn diagrams.
