# Supplementary figures and images for: Mapping the human oral and gut fungal microbiota in patients with metabolic dysfunction-associated fatty liver disease
Source: Front Cell Infect Microbiol. 2023 Apr 26;13:1157368. doi: 10.3389/fcimb.2023.1157368 (PMC10170973; doi:10.3389/fcimb.2023.1157368)

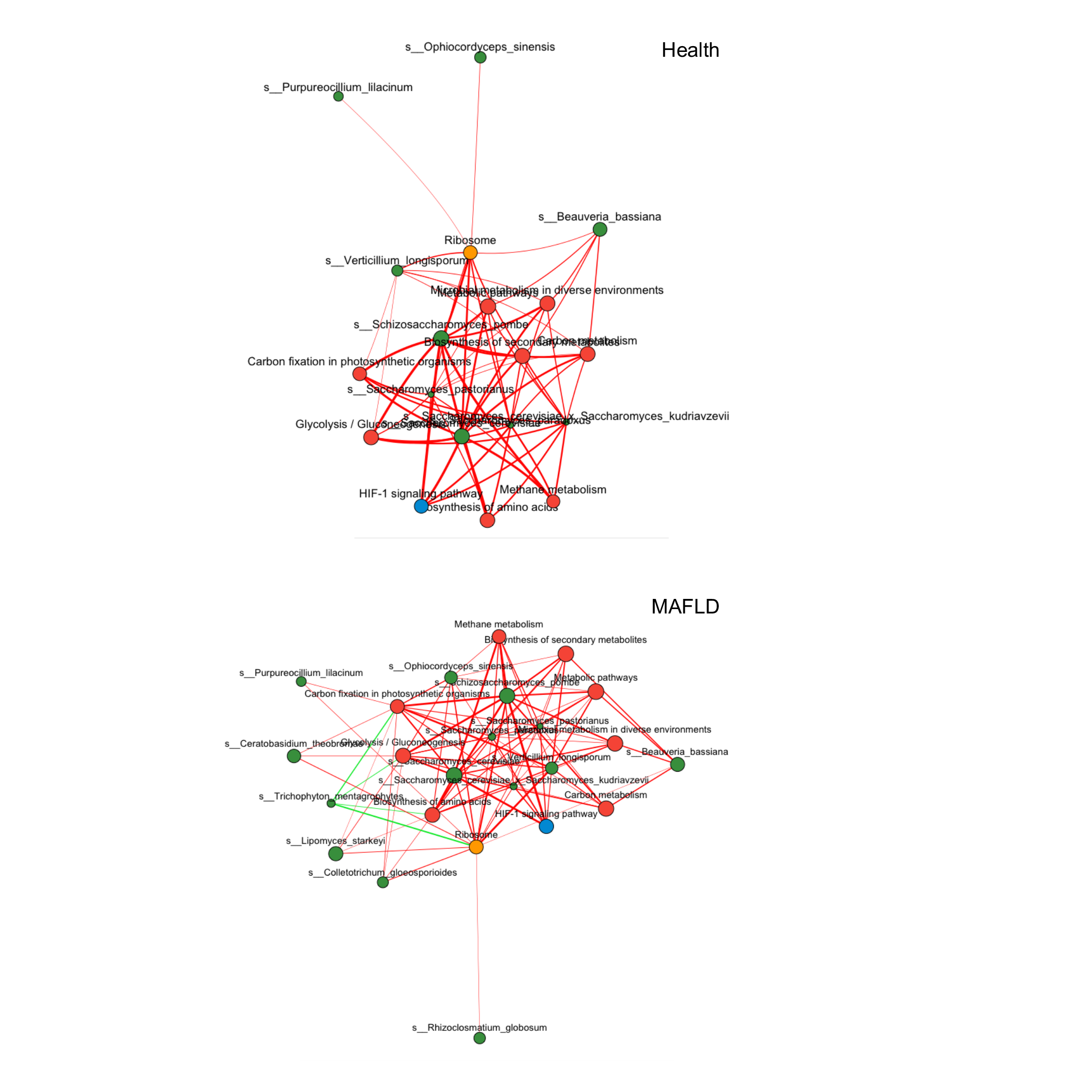

Supplement: Supplementary Figure 1 — Correlation analysis between function and fungal species of healthy controls and MAFLD patients. Correlation network analysis between functional alterations and differential fungal species. Spearman correlation > 0.5 or ≤ 0.5 is represented. [file Image_1.tif]

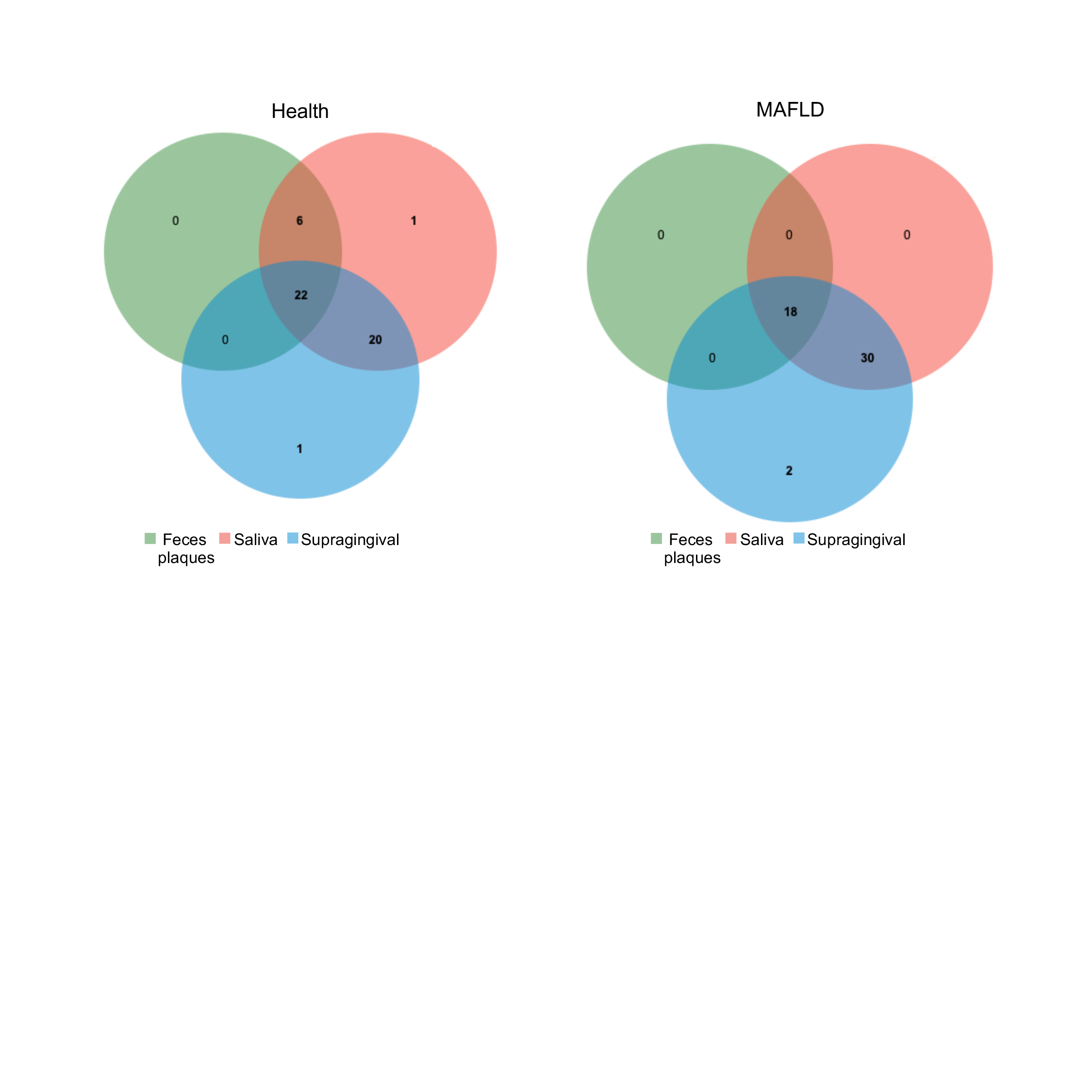

Supplement: Supplementary Figure 2 — Shared species in participants with or without MAFLD. Venn diagrams showing the unique and shared species among saliva, supragingival plaques, and feces. The 50 most abundant species were used for the Venn diagrams. [file Image_2.tif]
